# FunImageJ: a Lisp framework for scientific image processing

**DOI:** 10.1093/bioinformatics/btx710

**Published:** 2017-11-02

**Authors:** Kyle I S Harrington, Curtis T Rueden, Kevin W Eliceiri

**Affiliations:** 1 Computational and Physical Systems Group, Virtual Technology and Design, University of Idaho, Moscow, ID, USA; 2 Laboratory for Optical and Computational Instrumentation, University of Wisconsin-Madison, Madison, WI, USA; 3 Morgridge Institute for Research, Madison, WI, USA

## Abstract

**Summary:**

FunImageJ is a Lisp framework for scientific image processing built upon the ImageJ software ecosystem. The framework provides a natural functional-style for programming, while accounting for the performance requirements necessary in big data processing commonly encountered in biological image analysis.

**Availability and implementation:**

Freely available plugin to Fiji (http://fiji.sc/#download). Installation and use instructions available at http://imagej.net/FunImageJ.

**Supplementary information:**

Supplementary data are available at *Bioinformatics* online.

## 1 Introduction

Lisp programming languages have a long history in the development of computer programming. Lisp was the second high-level programming language to be developed, and has been one of the primary languages of artificial intelligence (AI). The utility of Lisp in AI is largely due to its capacity for expressing and manipulating code as data, allowing for functional programming styles. As AI becomes ubiquitous throughout the sciences, the need to incorporate machine learning and computer vision into the field of biological image analysis has become increasingly clear. To this end we have developed FunImageJ as a platform to facilitate the bridge between AI and the broadly used scientific image analysis platform, ImageJ. Recent efforts from the ImageJ community have vastly improved the accessibility and utility of free and open-access software tools for biological imaging for not only the user base in biological communities, but the software development community as well (Schindelin *et al.*, 2015). Many of these advances have been driven by the ImageJ2 and SciJava development thrusts, which have facilitated the development of declarative ImageJ2 plugins and the use of powerful ImgLib2 (Pietzsch *et al.*, 2012) data structures. While the ImageJ2 platform has brought many more developer tools and scripting languages to ImageJ, there has been no dedicated support for Lisp- and functional-style programming. Both programming paradigms offer a range of utility for image processing that derives first-class status of mathematical functions, the immutability of data, and treating code as hierarchical list structures. The application of mathematical functions to collections of pixels for image processing is intuitive to image analysts; however, the utility of immutability and code as hierarchies of lists may be somewhat unclear and could be seen as a limitation. Immutability prevents problems that arise from different software modules altering the state of the same data, which aids in the parallelization of processing routines; however, when dealing with big data, such as biological images, it is sometimes necessary to rely on mutable functions. For example, when implementing routines that operate on individual pixels in an image it is generally preferable to directly mutate the source image, as opposed to creating a duplicate image with a single altered pixel. In FunImageJ we attempt to maintain immutability up to the point of altering image data, at which point it becomes the user’s responsibility to duplicate image data if immutability is required. Finally, the representation of code as hierarchical list structures greatly facilitates metaprogramming techniques, allowing programmers to develop computer programs that generate image processing programs. It is this high-level capacity to use code to manipulate and synthesize programs that makes FunImageJ a powerful framework for the development of biological image processing software, which we demonstrate through the examples provided in this paper and Supplementary Material.

## 2 Materials and methods

FunImageJ was developed to both be used to create independently distributable programs, as well as ImageJ scripts that can be used within the Fiji distribution of ImageJ (Schindelin *et al.*, 2012). As ImageJ is primarily Java-based software, the underlying Lisp dialect is Clojure (Hickey, 2008), a Lisp built on the Java Virtual Machine. An initial implementation of an idiomatic Clojure framework for ImageJ (Harrington *et al.*, 2015) focused on providing accessors to ImageJ 1.x (Schneider *et al.*, 2012), including typing optimizations and some functional programming features. In FunImageJ, we incorporate a number of core features of the ImageJ2 (Rueden *et al.*, 2017), ImgLib2 (Pietzsch *et al.*, 2012), and ImageJ Ops to establish a powerful platform for image processing. The ImageJ Ops library provides a massive set of algorithms and operations for image processing, which FunImageJ dynamically discovers and re-encodes in idiomatic and optimized Clojure syntax. In terms of performance, most functions included in FunImageJ have the same performance as their Java counterparts; however, the dynamic typing nature of Clojure means that naïve algorithm implementations that do not provide data type information at appropriate times can suffer from performance penalties arising from automatic type inference. By providing a large collection of namespaces and functions, users can translate between images, meshes, ImageJ 1.x, ImgLib2 and ImageJ2 with ease. FunImageJ is open-source software under the permissive Apache 2.0 license.

## 3 Results

**Fig. 1:**
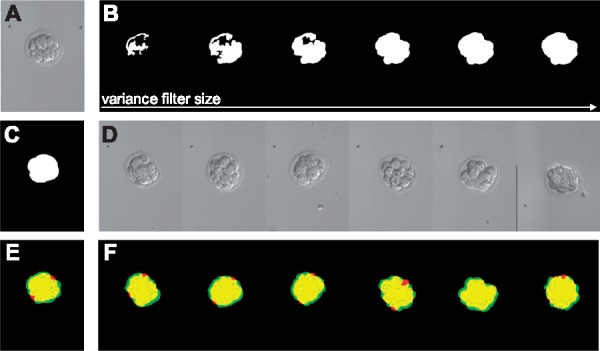
Optimizing detection of DIC imaged mouse embryos from a single image. (**A**) Test input, (**B**) candidate filters, (**C**) test target, (**D**) validation inputs, (**E**) overlaid target and detection show agreement for majority of embryo with minor disagreement at borders, (**F**) overlaid validation targets and detections

We present an example of FunimageJ applied to the BBBC003v1 image set from the Broad Bioimage Benchmark Collection (Ljosa *et al.*, 2012). The BBBC003v1 dataset contains a collection of 15 mouse embryos imaged using differential interference contrast (DIC), and a set of manually produced foreground segmentations. We present an algorithm can be concisely written that: filters particulates using ImageJ 1.x regions-of-interest, searches through a set of filters for the best match on one example image by maximizing segmentation accuracy, and applies the best filter to all candidate images. The results of this algorithm are shown in Figure 1.

## 4 Discussion

Most situations that warrant the use of FunImageJ will involve either a high degree of exploratory development or metaprogramming. Lisp dialects are traditionally developed iteratively using a ‘read-eval-print-loop,’ where the programmer slowly grows her code while ensuring accuracy throughout the entire development process. Additionally, the paradigm of code-as-data lends itself to writing code that uses functions as variables, making it easy to evaluate multiple variants of an algorithm with minimal alterations. While the dynamic discovery of algorithms provided via the ImageJ Ops library ensures that a subset of ImageJ algorithms are automatically incorporated into FunImageJ, on-going development efforts by the ImageJ community continue to introduce new algorithms and data structures (such as the upcoming ImgLib2 region-of-interest release, and ImageJ Tensorflow interface) and these advances will require future development to ensure that they are idiomatically incorporated into FunImageJ. By building upon ImageJ, FunImageJ benefits from the big data biological image processing capacity provided by ImgLib2 (Pietzsch *et al.*, 2012), KNIME Image Processing (Dietz and Berthold, 2016) and the ImageJ ecosystem (Schindelin *et al.*, 2015). As a result, FunImageJ has been able to focus on translating the benefits of Lisp programming to the world of biological image analysis.

## Supplementary Material

Supplementary Data
