# Supplementary material for: FunImageJ: a Lisp framework for scientific image processing
Source: Bioinformatics. 2017 Nov 2;34(5):899–900. doi: 10.1093/bioinformatics/btx710 (PMC6031065; doi:10.1093/bioinformatics/btx710)
Supplement: Supplementary Data [file btx710_supplemental_info_002.pdf]

Embryo Detection

Code is available online at:

https://github.com/kephale/fun.imagej/blob/master/test/fun/imagej/test/examples/BBBC003_detector.clj

```
(ns fun.imagej.test.examples.BBBC003_detector
  (:require [fun.imagej.img :as img]
            [fun.imagej.core :as ij]
            [fun.imagej.ops :as ops]
            [fun.imagej.img.shape :as shape]
            [fun.imagej.conversion :as convert]
            [fun.imagej.segmentation.imp :as ij1seg]
            [fun.imagej.imp.roi :as roi]
            [fun.imagej.img.type :as imtype]
            [fun.imagej.imp :as ij1]))

(defn extract-embryo
  "Extract an embryo parameterized by a radius."
  [input-img radius]
  (convert/imp->img; Return our result as IJ2 friendly
    (roi/fill-rois;  Fill in our detected ROI
      (convert/img->imp; Create an image for storing our ROI
        (img/create-img-like input-img))
      (take-last 1; Take the biggest ROI
         (sort-by roi/area; Sort the ROIs by area
              (ij1seg/imp-to-rois; Convert our image into ROIs
                (convert/img->imp; We need to process ROIs using IJ1
                  (fun.imagej.ops.threshold/isoData; Threshold using the isoData method
                    (fun.imagej.ops.filter/variance; Calculate a variance filter
                      (img/create-img-like input-img; Create an image for storing our filter
                                           (imtype/double-type))
                      input-img; The input image
                      (shape/sphere-shape radius)))))))))) ; Filter with a circular radius

(when false; Disabled, re-enable once you obtain the dataset from:
  ;            https://data.broadinstitute.org/bbbc/BBBC003/
  (let [bbbc-dir "/Users/kharrington/Data/BBBC/"
        input-imgs (let [input-dir (str bbbc-dir "BBBC003_inputs")]
                     (for [file (.listFiles (java.io.File. input-dir))]
                       (ij/open-img (.getPath file))))
        target-imgs (let [target-dir (str bbbc-dir "BBBC003_targets")]
                      (for [file (.listFiles (java.io.File. target-dir))]
                        (ij/open-img (.getPath file))))
        input-img (first input-imgs)
        target-img (first target-imgs)
```

```
   accuracies (sort-by second >;                         Sort our results by accuracy
                 (map (fn [radius]
                        (let [result-img (extract-embryo input-img radius)
                              cmat (img/confusion-matrix target-img result-img)]
                          [radius (float (:ACC cmat))]))
                      (range 10 25 3)))]
(println "Best result:" (first accuracies))

; Uncomment this if you want to see what the predictions look like
#_(doall
 (map (fn [target input-img]
        (ij1/show-imp
          (ij1/imps-to-rgb
            (map convert/img->imp; this can shrink
                 [target (extract-embryo input-img (ffirst accuracies))]))))
      target-imgs input-imgs)))
```
